# Case Investigation and Contact Tracing in US State and Local Public Health Agencies: Sustaining Capacities and Applying Lessons Learned From the COVID-19 Pandemic and 2022 Mpox Outbreak

**DOI:** 10.1089/hs.2023.0011

**Published:** 2023-09-27

**Authors:** Alexandra Woodward, Caitlin Rivers

**Affiliations:** Alexandra Woodward, DrPH, MPH, is a DrPH Researcher, Johns Hopkins Bloomberg School of Public Health and Johns Hopkins Center for Health Security, Baltimore, MD.; Caitlin Rivers, PhD, is an Assistant Professor, Department of Environmental Health and Engineering, Johns Hopkins Bloomberg School of Public Health, and a Senior Scholar, Johns Hopkins Center for Health Security, Baltimore, MD.

**Keywords:** COVID-19, Case investigation, Contact tracing, Public health preparedness/response, Infectious disease, Policy, Public health capacity

## Abstract

The COVID-19 pandemic illuminated the lack of resources available to US state and local public health agencies to respond to large-scale health events. Two response activities that were notably underresourced are case investigation and contact tracing (CI/CT), which health agencies routinely employ to control and prevent the transmission of infectious diseases. However, the scale of contact tracing required during the COVID-19 pandemic exceeded available resources, even in high-capacity public health agencies. For both routine outbreak response and epidemic preparedness, health agencies must have CI/CT program capacities in place prior to the detection of an outbreak to be ready to respond. Our research builds on previous work to identify the baseline CI/CT capacities needed in US state and local public health agencies to respond to any type of outbreak. Fifteen public health officials representing 10 public health agencies and 4 experts in CI/CT were interviewed about various aspects of their CI/CT program during the COVID-19 pandemic. The interviews coincided with the beginning of the 2022 mpox epidemic. Discussions on CI/CT during that response were collected to augment the interviews, where possible. Findings revealed that CI/CT capacities were underresourced prior to and during the pandemic, as well as during the mpox outbreak, even after substantial additional resourcing and efforts to scale up. Moreover, state and local health agencies encountered challenges in pivoting their COVID-19 CI/CT capacities for the mpox response, suggesting that CI/CT programs should either be designed with flexibility in mind, or should allow for specialization based on the pathogen's mode of transmission and the population at risk. Federal, state, and local health agency staff and officials should consider lessons learned from this research to plan for readily scalable and sustainable CI/CT programs to ensure readiness for future outbreaks.

## Introduction

Case investigation and contact tracing (CI/CT)* are fundamental public health strategies that are routinely employed to control and prevent the transmission of a variety of infectious diseases such as sexually transmitted infections, tuberculosis, measles, and for outbreaks of emerging infections such as Ebola.^1-3^ CI/CT strategies are broadly focused on promptly identifying cases and their contacts and applying prevention and control measures, thereby preventing further spread of a disease in a community.^4^ At the onset of the COVID-19 pandemic in the United States in January 2020, many state and local public health agencies had established CI/CT programs but lacked the baseline capacities needed to quickly expand their existing programs to respond to a wide-scale event. This may be, in part, because prior to the pandemic there were no comprehensive, disease-agnostic guidelines describing the scope of the capacities needed in state and local health agencies to conduct CI/CT.^2^

Lessons learned from previous outbreaks highlight the importance of implementing CI/CT at the early stages of an outbreak, when incidence is low, to rapidly break the growing chains of transmission.^3^ As the situation evolves, the objectives of CI/CT may need to adapt. As seen during the COVID-19 pandemic, CI/CT is challenging to maintain during periods of high transmission because the volume of cases and contacts that require follow-up exceeds the capacity that even a well-staffed workforce can handle. Therefore, it is critical for state and local health agencies to have scalable baseline CI/CT capacities in place prior to the detection of an outbreak to ensure that these programs are ready to respond to emerging outbreaks.

To address the lack of comprehensive guidelines describing the capacities and capabilities of CI/CT programs, our earlier research identified and described the relationships between these capacities and capabilities.^5^ Capacities refer to the organizational, technical, and social resources (eg, governance, workforce, technology) that are needed to support a CI/CT program's capabilities. Capabilities are aligned with the goals of a CI/CT program and describe what the program is able to accomplish. To date, there has been little guidance around the minimum, baseline capacities that should be maintained in state and local public health agencies on a continuous basis to ensure that programs are able to conduct both routine CI/CT and maintain readiness to scale up these programs during large outbreaks.^5^

Several studies have explored lessons learned around conducting CI/CT during the earlier stages of the COVID-19 pandemic.^2,6-11^ However, few publications include a review of knowledge that public health agencies gained while implementing, sustaining, or scaling baseline CI/CT capacities at the state and local levels over the course of the COVID-19 pandemic, and during the 2022 mpox outbreak in the United States. Our study builds on earlier research to identify and characterize the CI/CT capacities that should be maintained as baseline functions in state and local public health agencies on a continuous basis to ensure that health agencies are prepared to (1) conduct CI/CT in future outbreaks, (2) identify lessons learned from scaling up CI/CT capacities during the COVID-19 pandemic as well as during the 2022 mpox outbreak, and (3) identify gaps in CI/CT capacities that continue to exist today. Findings from this research may inform senior public health officials and public health department leadership in building and sustaining CI/CT programs to respond to future outbreaks.

## Methods

Our earlier research described a narrative literature review to identify the capacities of CI/CT programs.^5^ Capacities were bucketed into 3 broad domains—organizational, technical, and social—based on the nature of their function. Once this CI/CT capacity framework was developed, we conducted qualitative interviews between May and August 2022 with officials at US state and local health agencies and public health experts in federal agencies and public health organizations.

### Inclusion Criteria and Participating Public Health Agency Selection

Participating state and local public health agencies were defined as those that conducted CI/CT during the COVID-19 pandemic. Public health experts were defined as those who informed CI/CT strategies during the pandemic. Potential key informants from state and local public health agencies were identified using a 2-part process: first, their agencies had to meet specific criteria based on their agencies ability to conduct CI/CT, and second, the potential interviewees had to be senior-level staff who managed CI/CT programs.

State and local public health agencies were selected using on 2 primary factors: (1) workforce capacity (whether the agency met the recommended benchmark of 30 public health professionals per 100,000 population to conduct CI/CT) and (2) CI/CT program model type (in-house, contracting, and/or partnering) used by the agency as of December 2020.^12,13^

The first factor (workforce capacity) was chosen based on a report published by the National Association of County and City Health Officials in April 2020 recommending that 30 contact tracers per 100,000 population, distributed across health agencies in an equitable manner (eg, using a per capita formula), were needed to effectively conduct CI/CT during the COVID-19 pandemic.^14^ Using this benchmark, potential state agency interviewees were invited to participate based on eligibility as determined by a review of data collected in a December 2020 CI/CT workforce survey by the Johns Hopkins Center for Health Security and National Public Radio.^15^

The second factor for key informant selection was the CI/CT program type (in-house, contracting, and partnering) used by their agency. An in-house model involves state and local officials who lead the CI/CT programs, hiring or recruiting volunteers as needed. In a contracting model, the state contracts with an organization for CI/CT hiring and relies on the organization for training/staffing. Finally, in a partnering model, the state leads the efforts but relies on partners for training/staffing. All 3 models were represented in the interviews, with many states combining models during the pandemic.^16^

Additionally, we aimed to have a final study population that represented both US political parties (represented by the state governor's political party as of December 2020), all US census regions, and a variety of high- and low-population densities across the country (based on 2022 US census data).

### Key Informant Selection

We recruited key informants via outreach supported by a nonprofit organization that works to advance public health in state and local jurisdictions. The organization provided contact information for all agencies that met our CI/CT and population requirements and we sent a recruitment email with background information about the study to a total of 7 state-level public health agencies. Senior-level CI/CT managers from 5 agencies agreed to participate. Of these, 3 facilitated recruitment to 1 or 2 local health agencies within their state to participate in interviews as well. This resulted in a total of 15 interviewees from 10 state and local agencies. Additionally, we used a snowball sampling method to identify public health experts in CI/CT from several public health partner organizations and US Department of Health and Human Services agencies to participate in individual interviews. Four people agreed, bringing the total study population to 19.

The Johns Hopkins Bloomberg School of Public Health Institutional Review Board reviewed and designated study protocol (IRB No. 00019635) exempt and not human subjects research.

### Interview Guide Development and Data Analysis

We developed a semistructured interview guide to facilitate discussion. Each interview lasted 1 to 2 hours. Key informant interviewees received read-ahead materials, which included the CI/CT capacity framework (shown in Table 1) identified in the narrative review of literature. Interviewees were queried on capacities that are critical to maintain at baseline in public health agencies, lessons learned around scaling up and maintaining capacities during the COVID-19 pandemic, and lessons from the 2022 mpox outbreak. The study team recorded and transcribed all interviews, and the transcripts were reviewed and coded by 1 researcher with deductive approaches using NVivo version 11 (QSR International, Burlington, MA). We conducted a thematic analysis to identify the capacities discussed during the interviews.^5^

**Table 1.** Baseline CI/CT Program Capacities Identified During Interviews

Domain	Capacity	No. Expert Interviewee Mentions (n=4)	No. State Health Agency Interviewee Mentions (n=5)	No. Local Health Agency Interviewee Mentions (n=10)	Total Interviewee Mentions (N=19)
Organizational	Governance	2	1	1	4
	Funding^a^	4	4	7	15
	Partnerships	0	1	2	3
	Workforce	4	3	8	15
Technical	CI/CT processes, protocols, and forms	1	0	1	2
	Data collection, management, and analysis systems	3	4	4	11
	Digital and technology solutions	0	0	0	0
	Privacy and data sharing	0	0	0	0
	Metrics and monitoring^b^	0	0	2	2
	Clinical consultation	0	0	1	1
	Procurement and distribution of materials	0	2	1	3
	Out-of-home isolation/quarantine	0	1	0	1
Social	Wraparound services	1	0	1	2
	Public communication and engagement	2	1	2	5

^a^ Funding was not included in the capacity list at the time of the interview but was added as a capacity based on discussions.

^b^ Metrics and monitoring capacity was moved from the organizational to the technical domain based on interview responses.

Abbreviation: CI/CT, case investigation and contact tracing.

## Results

### Key Informants

We conducted semistructured interviews with 19 key informants: 15 senior-level public health staff who represented 10 participant state and local health agencies that were involved in CI/CT programs during the COVID-19 pandemic (Table 2) and 4 CI/CT experts from public health partner organizations and US Department of Health and Human Services agencies. Five of the 10 public health agencies met the workforce benchmark of 30 tracers per 100,000 population and 5 did not meet this benchmark. All participating state agencies used a mix of program models (in-house, contracting, and/or partnering), except 1, which relied solely on the in-house model.

**Table 2.** Characteristics of the 10 Participating Public Health Agencies

State Participant	Health Agency Type	Met 30/100K Tracer Benchmark?^a^	Program Model (In-House, Contracting, Partnering)^b^	Governor's Political Party	US Census Region^c^	Population Density (Low/High)^d^	No. of Interviewees (N = 15)
A	State	No	In-house, contracting	Democrat	South (West South Central)	Low	2
B	Local^e^	Yes	In-house, contracting	Democrat	Northeast (Middle Atlantic)	High	2
C	State	No	Partnering, contracting	Republican	South (South Atlantic)	High	1
D	Local	No	Partnering, contracting	Republican	South (South Atlantic)	High	1
E	State	Yes	In-house, contracting	Republican	Northeast (New England)	High	1
F	Local	Yes	In-house, contracting	Republican	Northeast (New England)	High	2
G	Local	Yes	In-house, contracting	Republican	Northeast (New England)	High	1
H	State	No	In-house	Republican	West (Mountain)	Low	1
I	Local	No	In-house	Republican	West (Mountain)	Low	1
J	Local	No	In-house	Republican	West (Mountain)	Low	3

^a^ Met the workforce benchmark of 30 tracers per 100,000 population,^14^ based on data collected by the Johns Hopkins Center for Health Security and National Public Radio in December 2020.^15^

^b^ Based on program model type identified by the National Academy for State Health Policy as of December 2020.^13^

^c^ Based on US census regions.^16^

^d^ Based on US census data from 2021; “low” refers to population densities below the average (208.86 people/mi^2^) and “high” refers to population densities above the average.^17^

^e^ Not a health agency, but a separate program contracted to conduct case investigation and contact tracing for an entire major metropolitan city in collaboration with the local health agency.

### Baseline Capacities

Most key informants recognized that all capacities listed in the CI/CT capacity framework are important to maintain as baseline public health functions. However, all key informants noted that certain capacities are more important to maintain than others. The capacities discussed most frequently by all key informants were funding, workforce, and data collection, management, and analysis systems (Table 1). Lessons learned and major themes discussed around these most frequently discussed capacities, as well as a few others, are included as follows.

### Workforce

When asked which capacities are critical for CI/CT programs to function during outbreaks, all 19 interviewees noted that an impactful CI/CT program is dependent on a sufficiently staffed and trained workforce that can build trust among diverse communities within the population. Many felt that their agency maintained a trained workforce prior to the pandemic, but that existing capacities were not sufficient to meet CI/CT needs during the pandemic. The key informants made several observations of relevance to further the design and maintenance of scalable CI/CT programs.

#### Workforce Hiring Approaches

Seven of the 10 participating health agencies used contracted staff to expand CI/CT capacities at some point during the pandemic. This approach enabled rapid scale-up of the workforce, but it presents challenges and does not result in a sustainable in-house (health agency) public health workforce. Specifically, the 4 state or local health agencies that met or exceeded the workforce benchmark of 30 tracers per 100,000 population all employed the mixed in-house/contracting model. Despite having met the benchmark, these agency managers reported having an insufficient number of CI/CT staff during the pandemic. They discussed how the contracted workforce “roller coastered” as investigators and tracers were hired and let go during the peaks and valleys of the pandemic. This unsteady, unpredictable workforce complicated planning for in-house workforce needs and responsibilities, as program managers were often unaware of how many contractors were available for support.

Agencies that did not meet the workforce benchmark prioritized expansion of in-house staff, particularly after funding became available through the Coronavirus Aid, Relief, and Economic Security (CARES) Act of 2020^18^ and American Rescue Plan Act of 2021^19^ to support hiring actions. One interviewee marveled at the agency's ability to scale up their in-house CI/CT workforce from zero to 120 staff between January and August 2020 using funds provided by the CARES Act. Still, these key informants emphasized a shortage in trained and available staff that resulted in insufficient capacity.

Reflecting on approaches taken during the pandemic, interviewees offered a few recommendations for scaling CI/CT workforce during large-scale outbreaks. First, several interviewees discussed the need for state and local health agencies to maintain a sizable cadre of permanent in-house staff while also planning for a reserve workforce capable of conducting CI/CT. Second, 2 of the 19 interviewees (including the CI/CT experts) urged the US Centers for Disease Control and Prevention (CDC) to maintain surge capacity that can be deployed to health agencies in times of need. These recommendations are not necessarily mutually exclusive and may be considered in layered and overlapping approaches.

#### Workforce Skills

Interviewees conveyed a variety of perspectives regarding the appropriate skills of a CI/CT workforce; 16 interviewees expressed being confronted with the tradeoff between having a workforce that can handle a high volume of cases and contacts at a rapid pace and taking more time during interviews to provide tailored social support and build rapport. The mention of this balance resulted in further discussions around what a CI/CT program should be capable of accomplishing. All 19 interviewees expressed the sentiment that to build trust with the affected community, CI/CT staff need to be culturally and linguistically appropriate for the communities they serve. However, there was a noted discordance in responses about other skills. Six interviewees valued a workforce that can reach the maximum number of cases and contacts, which they felt was best accomplished by closely following an interview script. This approach generally precludes off-script conversation. This type of workforce may not need deep expertise in public health or nuanced interviewing skills.

On the other hand, 4 interviewees discussed the importance of connecting with individuals on a deeper level using a semiscripted approach. Flexible conversations enabled investigators and tracers to address specific needs in the people they reached, such as arranging for medication, food, and emotional support. This approach also allowed for tracers to answer health questions not related to COVID-19, such as how to sign up for Medicare. Tracers staffing a workforce using this broader approach should be trained in introducing empathy into their interviews. These individuals may include health professionals like nurses and social workers with expertise in basic medical questions, social support, and safety net programs. A workforce with these skills may be more capable of building a rapport and gaining trust with communities. For example, 1 interviewee, a nurse who ran the CI/CT program in a small town, frequently went off-script to have a more empathetic conversation with individuals who expressed grief and mistrust. She felt this enabled her to build trust and facilitate an understanding of isolation and quarantine procedures and resources. Another interviewee discussed how CI/CT outreach was a “lifeline” for those navigating the pandemic alone. The key informant valued going off-script to share tailored resources that individuals could leverage to return to their health and lifestyle.

Several interviewees discussed the value that clinical skills may bring to CI/CT programs, yet not all agreed that clinical staff, such as nurses, are needed for conducting CI/CT. Notably, there was a discrepancy in perspectives between 1 state health agency interviewee and 2 of the local health agency interviewees in that same state: the state interviewee discussed spending a “significant portion” of workforce funds on hiring nurses to conduct CI/CT, yet clinical skills were not needed to conduct CI/CT. However, 2 local interviewees discussed the value that nurses and social workers bring with their empathetic interviewing skills.

Finally, the key informants emphasized that it is critical for health agencies to consider how tracers are trained and experienced in protecting data privacy, including protected health information and personal identifiable information. Seven interviewees highlighted the need for a CI/CT workforce to be trusted with handling this information. The key informants whose state relied on the contracting model more frequently discussed challenges related to trust in contractors protecting health information and personal identifiable information, despite going through the required Health Insurance Portability and Accountability Act^20^ training. One interviewee hypothesized that tracers without a background in public health or healthcare may not understand the importance of protecting this information, so hiring individuals with these backgrounds should be prioritized.

#### Programmatic and Supervisory Staff

Programmatic and supervisory skills are critical to maintain among health agency staff to ensure the ability to oversee CI/CT programs during outbreaks. Several interviewees discussed the need for more supervisory staff to direct the operations of CI/CT programs, as this type of staff was lacking during the COVID-19 pandemic, resulting in challenges such as delays in hiring and training the CI/CT workforce. These staff should be capable of conducting hiring and training and ensuring quality of the workforce, developing and maintaining protocols, evaluating CI/CT data, and strategizing community engagement. A few interviewees had enough investigators and tracers but lacked sufficient staff to oversee the operations of the program.

#### Compensation, Benefits, and Mental Health Support

CI/CT staff need appropriate compensation, benefits (including counseling and sufficient vacation days), and recognition of the value of their work. All 19 interviewees discussed burnout and declining morale among the CI/CT workforce as the pandemic progressed. These comments were particularly emphasized among local public health key informants, who noted that attrition was high because of insufficient compensation and a lack of resources to help staff recover from burnout.

### Data Collection, Management, and Analysis

CI/CT relies on data collection and management systems that are capable of ingesting multiple data streams, such as data from case and laboratory reporting. Several interviewees emphasized that these systems should be affordable, nimble, and integrated with the data collection approach (eg, voice/telephone), and should contain data collection scripts and packages that can be rapidly updated by CI/CT program staff. Seven of the 10 health agencies interviewed (a total of 4 of the 5 states represented) contracted with Salesforce during the pandemic to implement a CI/CT software platform, replacing outdated, manual approaches that were insufficient for capturing the high volume of COVID-19 CI/CT data.

While many of the interviewees found that the Salesforce platform greatly improved upon existing processes, there was room for improvement. Despite its rapid implementation, Salesforce was expensive for many states. Public health staff were unable to adjust the platforms themselves, having to rely on information technology staff from the state's office of technology and the Salesforce team to make platform updates (eg, question packages). As Salesforce did not completely meet the needs of many local health agencies, staff often relied on additional tools such as spreadsheets, particularly when the state health agency was not able to assist in updating the platform due to resource constraints. Reliance on multiple data systems is cumbersome and leaves room for error in data entry and management.

To address the challenge of rapidly developing and deploying CI/CT data systems at the onset of a health event, an interviewee recommended that the federal government should support the development of CI/CT information systems that can be used for any disease requiring CI/CT. This may include a range of different software options that meet a baseline set of minimum requirements; health agencies can select the tailorable software (without assistance from the developer) that meets their specific needs. Several interviewees discussed specific requirements for such a platform. First, the platform should be interoperable with other relevant data systems within the state, such as electronic case, laboratory, and immunization reporting systems, so data can be easily exchanged between systems. Second, it should be coupled with telephone software, with data being captured as staff are speaking to cases and contacts, and should incorporate built-in branching logic that enables access to the appropriate question packages as interviews progress.

Finally, a few interviewees discussed a lack of basic technology and broadband access to support their CI/CT programs. Local health agency key informants discussed broader technology challenges more frequently than state key informants, such as the need for more functional computers, telephones, and broadband coverage in local areas where staff conducted CI/CT remotely from home.

### Funding

Many interviewees noted that state health agencies did not have sufficient funds to scale up CI/CT program capacities at the beginning of the COVID-19 pandemic. Several emphasized that health agencies need permanent, flexible funds provided by mandatory federal spending that can be used without a public health emergency declaration to be able to maintain CI/CT capacities at baseline. As local health agencies are primarily responsible for conducting CI/CT in many scenarios, sufficient funds for CI/CT capacities should be appropriated on a consistent basis. While the CARES Act provided funds to support initial scaling, 3 interviewees experienced months-long delays in receiving funds, particularly at the local level. At the onset of the pandemic, states relied on contracts with external vendors because they did not have sufficient funds or authority to hire new staff. This reliance on contracts hindered public health agencies in developing a sustainable in-house workforce.

While 9 state interviewees discussed having received sufficient funding from the federal government once the CARES Act and American Rescue Plan Act funds were dispersed in 2020 and 2021, many local interviewees reported being underfunded and lacking clear guidance from the state health agency about how to spend the funds. For example, 2 local key informants discussed a lack of clarity about whether and how funds could be used for wraparound services (eg, food, medication delivery, paid sick leave); staff paid for food and other supplies for those in isolation and quarantine out of their own pockets.

### Partnerships

Several interviewees emphasized the importance of partnerships with community-based organizations (CBOs), schools, and social services to connect with diverse communities. One interviewee recommended that federal grants require health agencies to demonstrate sustained relationships with communities, such as via contracts with CBOs. Local health agency interviewees more frequently discussed how their partnerships were strengthened during the pandemic than did state-level interviewees. A few spoke to the value of grassroots approaches in improving community responsiveness to CI/CT outreach. For example, an interviewee discussed the establishment of a community-based outreach team during the pandemic, staffed by paid high school students from the community who went door-to-door in hard-to-reach communities with information and resources to prevent transmission and encourage responsiveness to CI/CT outreach. While the impact of this outreach team was not quantitatively measured, the interviewee discussed anecdotal evidence of increased participation in CI/CT among the community after the outreach began.

Additionally, key informants encouraged strong partnerships between health agencies and healthcare providers in the community. Several discussed how healthcare providers can assist with timely notification of suspect cases to the health agency when laboratory results are delayed; communicate information about the disease and prevention measures to patients; and alert cases that they will be contacted by the health agency, encouraging them to “answer the call.” Levels of willingness to share information with public health authorities during CI/CT outreach and to isolate and quarantine have been shown to be higher when the request to do so comes from healthcare providers compared with federal public health authorities. Health agencies should continue to build partnerships with healthcare providers in their communities, as providers can enable trust between their patients and public health, improving participation in CI/CT.^21^

Another theme that emerged from the interviews was the importance of sharing capacities—such as CI/CT workforce and protocols, processes, and forms—between state and local health agencies and across state lines. State and local health agencies held recurring meetings during the pandemic to discuss changing CI/CT guidance and approaches. These engagements strengthened relationships between health agencies, which led a state to develop agreements across local health agencies so CI/CT capacities can be shared across jurisdictions. Many interviewees noted that these strengthened relationships will likely be sustained beyond the COVID-19 pandemic.

Finally, many interviewees noted a need for stronger cross-state collaboration on pandemic response more broadly (beyond CI/CT). The Council for State and Territorial Epidemiologists held monthly meetings for state and local health agency staff to discuss CI/CT practices; CDC staff joined these meetings to communicate their agency's guidance. Interviewees discussed how further opportunities for cross-state collaboration should be explored during pandemic peacetime so partnerships are in place prior to future outbreaks.

### Public Communication and Engagement

To better communicate, engage, and build trust with communities, health agencies should develop a shared language around CI/CT that resonates with communities and should offer an outcome that benefits individuals when conducting CI/CT. To develop a shared language, an interviewee recommended the use of alternative terms such as “COVID-19 care specialist” or “community outreach specialist,” as the term “contact tracing” may connote ideas of government surveillance and invasion of privacy.^22^ The same interviewee emphasized that future CI/CT programs must clearly define what a program can offer to individuals rather than what they may perceive as government surveillance. A few other interviewees echoed this sentiment as they discussed the importance of CI/CT offering an outcome or service to individuals, such as access to prevention (eg, personal protective equipment, vaccines), treatment, wraparound resources, and actionable information at the individual level.

### Lessons From Pivoting COVID-19 CI/CT Capacities to Mpox Response

At the time of the interviews, 8 key informants were conducting CI/CT in response to the 2022 mpox epidemic. Where relevant, interviewees were queried on whether CI/CT capacities employed for COVID-19 were being leveraged for mpox. Themes from these discussions are described as follows.

#### Workforce

Public health agency key informants had mixed perspectives about whether they had sufficient CI/CT staff to respond to mpox. While some primarily relied on existing internal health department staff and felt that was sufficient, others were frustrated that they could not leverage the staff they hired for COVID-19 CI/CT due to funding constraints because funds used for the COVID-19 workforce could not be used for other diseases. Several interviewees emphasized that future supplemental funding for CI/CT should be flexible enough to contribute to control of other infectious diseases.

#### Data Collection, Management, and Analysis

Three interviewees reported that they did not use dedicated data systems (such as Salesforce) that were deployed for COVID-19 (including 3 of the 4 states that used Salesforce in response to COVID-19). Instead, some interviewees discussed leveraging “simpler” systems in use prior to COVID-19 because they could be adapted more rapidly for mpox. Many felt that adapting Salesforce for mpox would take too much time and too many resources. Some states reported using shared spreadsheets to collect and manage CI/CT data for mpox, which posed challenges in terms of efficiency and data quality. Additionally, several states implemented text message (SMS) approaches during the pandemic to monitor symptoms more efficiently among cases and contacts; a few of these states continued this practice for mpox, which has anecdotally saved time and resources.

#### Wraparound Support Services

Two interviewees reported that limited funds hindered their ability to provide wraparound services for mpox cases and contacts. Requests from some communities for wraparound services could not be met due to funding constraints.

## Discussion

While the key informants confirmed that all capacities are critical for effective CI/CT programs, the most frequently discussed capacities as baseline functions were funding; workforce; and data collection, management, and analysis systems. These may be prioritized in near-term capacity building, as they were vastly underresourced and insufficient during the pandemic.

### Workforce

All key informants expressed the sentiment that state and local health agencies need more permanent staff trained on CI/CT, but many offered different perspectives on CI/CT workforce models. The “roller coaster” approach of bringing on contract staff for short periods often does not enable the maintenance of CI/CT institutional knowledge in a state; skills are lost if staff are let go at the end of an outbreak and do not return to the public health workforce. Funding for a permanent workforce trained in CI/CT in state and local health agencies may never be sufficient to meet the needs of a pandemic response. However, states should consider other approaches, such as a combination of permanent in-house staff and a reserve community-based workforce that receives annual refresher training on CI/CT, which has also been described by public health organizations and federal agencies.^23,24^

### Data Collection, Management, and Analysis Systems

States employed patchwork approaches to collecting, managing, and analyzing CI/CT data. The federal government should support the development of CI/CT systems that can be leveraged for any disease: this recommendation should be considered in light of CDC's recent Data Modernization Initiative,^25^ which may enable the development of such systems. CI/CT data systems should meet a baseline set of minimum requirements, including the ability to be interoperable with other health data systems operated at the federal and state levels, allowing for the integration of case (eg, clinical), laboratory, and immunization data, and should follow standards currently being developed such as the United States Core Data for Interoperability.^26^ Improved data integration will enable more timely, complete, and robust analyses of CI/CT data to inform public health decisionmaking.

### Funding

Federal and state agencies should consider updated approaches to distributing funds to local health departments in a timely manner. For example, CDC may consider directly distributing funds to local jurisdictions and/or requiring states to share a certain proportion of funds with local health departments (the latter is an approach currently being taken by CDC through the 2022 Public Health Infrastructure Grant Program).^27,28^ Moreover, state health agencies need to provide clear guidance around how funds can be used for CI/CT capacities, as a few local health agency interviewees noted a lack of clarity in the state health department's guidance for capacities such as wraparound services.

### Limitations and Future Research

The study population was not representative of all US health agencies; however, key informants from diverse settings guided by the selection criteria provided a range of perspectives. The quantification of themes discussed in the Results section are intended to represent the study population rather than the broader US public health system. While we aimed to obtain granular estimates on funds needed to support specific CI/CT program capacities (as compared to general outbreak response funds), many interviewees were unable to provide funding amounts that would inform estimates needed for each capacity. Future research around funding should include input from those responsible for managing budgets in health agencies. Finally, themes focused on pivoting CI/CT programs to the 2022 mpox response should be explored again in the future, as the interviews for this study took place between May and August 2022 when the outbreak had not yet reached its peak in many states.

## Conclusion

Several interviewees mentioned the analogy of “building a plane while flying it” when discussing CI/CT during the COVID-19 pandemic. The identification of baseline capacities needed to prepare a public health agency to conduct CI/CT is the first step toward building and sustaining these capacities. This research revealed that CI/CT capacities were underresourced both prior to and during the pandemic, as well as during the mpox outbreak in 2022, and more effective strategies could be considered to implement and sustain CI/CT programs. Further engagement of federal, state, and local health agency staff and officials, as well as public health partner organizations, is needed to strategize the future of CI/CT programs based on lessons learned from this research.

## Notes

^*^
While these processes can be conducted separately with distinct goals and benefits (eg, case investigation is sometimes conducted without contact tracing), our research refers to these processes as a concept with the abbreviation “CI/CT.”
